# Growth hormone – releasing hormone in the context of inflammation and redox biology

**DOI:** 10.3389/fimmu.2024.1403124

**Published:** 2024-06-18

**Authors:** Agnieszka Siejka, Nektarios Barabutis

**Affiliations:** ^1^ Department of Clinical Endocrinology, Medical University of Lodz, Lodz, Poland; ^2^ School of Basic Pharmaceutical and Toxicological Sciences, College of Pharmacy, University of Louisiana Monroe, Monroe, LA, United States

**Keywords:** tumors, redox biology, endothelium, lungs, hypothalamus, pituitary

## Introduction

Inflammation and oxidative stress contribute in cancer development, severity and aggression (1–3). Many malignancies arise from tissues affected by chronic inflammation. Tumor microenvironment consists of both cancer and immune cells which secrete growth factors, cytokines and chemokines, leading to cancer spread (1). Anti-inflammatory and immunomodulatory therapeutic approaches are commonly used in oncology (3).

Reactive oxygen species (ROS) are highly active molecules, arising from physiological and pathological processes, and our body balances their excess utilizing anti-oxidative defense mechanisms. Cancers suppress antioxidative mechanisms via enzyme modulation/mutation. Under physiological conditions ROS act as signaling molecules in cell growth, migration and differentiation. Chronic inflammation may lead to the excessive generation of ROS and reactive nitrogen species (RNS), altering immune responses, which in turn lead to oncogenic transformations (4).

The innate immune system depends on ROS, since macrophages and natural killer (NK) cells utilize those highly active molecules to maintain human tissue integrity and combat pathogens. ROS generation by mitochondria is due to activation of several proinflammatory pathways (e.g. MAPK, AMPK, PI3K/ACT) in coordination with NF-κB and HIF1α (2).

## Growth hormone releasing hormone and its receptors

Growth hormone - releasing hormone (GHRH) is secreted by the hypothalamus and binds to the GHRH receptor (GHRH-R) of the pituitary cells to trigger the release of GH from the somatotrophs. GHRH is a 44-amino acid peptide, however its full intrinsic biological activity is retained by the NH_2_-terminal 29-amino acid sequence. The pituitary type GHRH receptor (pGHRH-R) is a class II G-protein-coupled receptor with seven transmembrane domains, homologous to the receptors for VIP, PACAP and calcitonin. Activation of pGHRH-R results in increased cAMP production, which acts as the second messenger in the GHRH related signal transduction. Splice variants (SVs) of the GHRH-R have been identified in various cancers. SV1 receptor possesses ligand independent activities (5, 6) and activates the mitogen-activated protein kinase (MAPK) pathway (5, 7).

## GHRH in inflammation and tumors

The expression of GHRH has been demonstrated in prostatic, endometrial, ovarian, breast, gastroenteropatic, and lung carcinomas, glioblastomas, malignant bone tumors, human adrenal carcinomas and colorectal cancers (8). GHRH may act as an autocrine and/or paracrine growth factor in cancers (8). Knocking down of GHRH gene expression suppressed the proliferation of T47D, MDA-MB-435S, MDA-MB-468 breast cancers, LNCaP prostate cancer and NCI H838 non-SCLC (6). Moreover, GHRH can increase IL-17 secretion (9), a cytokine involved in the pathogenesis of non-alcoholic and alcoholic steatohepatitis (10). It has been reported that both conditions are associated with increased risk of hepatocellular carcinoma (HCC) development. In mice, targeting IL-17 suppressed the development of NASH-associated HCC (10). In another study, IL-17 was able to blunt the anticancer efficacy of chemotherapeutic agents *in vivo* (11). GHRH was able to promote TH17 cell differentiation and autoimmune inflammation (12), and MIA-690 – a GHRH antagonist - inhibited LPS-induced inflammatory and pro-oxidative markers (13).

Several splice variants of the GHRH receptor (SVs) were identified and sequenced, including SV1 (14). The major part of its cDNA sequence is identical to the corresponding sequence of pGHRH-R, with the exception of the first 334 SV1 nucleotides. The protein sequence of this transduced receptor differs from the full length receptor in the amino-terminal extracellular domain, in which a 25 amino-acid sequence replaces the first 89 amino acids of pGHRH-R (14). SV1 has been associated with strong ligand independent activities (15). Moreover, it is expressed in many cancers, including prostatic, breast, colorectal, gastric, melanomas, bone sarcomas, glioblastomas and SW13 human adrenal carcinoma cells (16). The pGHRH-R is present in human cancer tissues isolated from breast, ovarian, lung cancers, glioblastomas and lymphoma cells (16).

## GHRH antagonists in cancers

Antagonists of growth hormone-releasing hormone inhibit the growth of various experimental cancers including prostate, breast, ovarian, colorectal, lung, renal, endometrial cancers; glioblastomas and lymphomas (16, 17). The inhibitory effect of GHRH antagonists is partially dependent on the suppression of GH secretion from the pituitary, which results in decreased IGF-I production. GHRH antagonists can also suppress tumor growth in a direct manner through blockade of autocrine GHRH action (8, 16). HeLa cells, which do not express GHRH receptors, responded to GHRH and GHRH antagonists after being transfected with the pGHRH-R or SV1 receptor (18).

## GHRH and ROS

Reactive oxygen species (ROS) and reactive nitrogen species (RNS) act as signaling molecules. They promote human tumors by contributing to oxidative stress, a common condition in cancer (2). In LNCaP prostate cancer cells GHRH antagonists exerted antioxidative properties, and in A549 lung cancer cells JV-1–36 suppressed hydrogen-peroxide induced ROS (19, 20). In bovine pulmonary artery endothelial cells, human cerebral microvascular endothelial cells, and human lung microvascular endothelial cells those peptides reduced ROS generation. 3T3 cells which do not express GHRH receptors were not affected by GHRH analog treatment (21).

It was also recently revealed that GHRH antagonists suppress IFN-γ (22), hydrogen peroxide (23, 24) and hydrochloric acid (25) - induced inflammation. P53 is a tumor suppressor exerting anti-oxidative activities, which is induced by GHRH antagonists (17, 26–29). Those data further our knowledge on the mechanisms mediating the protective effects of those peptides against human disease (30). P53 and unfolded protein response are interrelated in the intracellular niche, since UPR activation induces P53 (31). It appears that UPR – which exerts anti-inflammatory and anti-oxidative activities (32–37) - is involved in the effects of GHRH antagonists in endothelial cells (32). These peptides were able to induce the three UPR sensors and its downstream target, namely BiP, in normal lung cells. There is very limited information on these effects of GHRH-related analogs in cancer cells (38).

It has been demonstrated that the SV1 receptor and pGHRH-R activate mitogen activated protein kinases ERK1/2 (7), which are strongly related to the generation and metabolism of ROS. GHRH can also activate: i) JAK2/STAT3, which contributes to oxidative phenomena (39), and ii) inducible nitric oxide synthase (iNOS) in A549 lung cancer cells. GHRH antagonist treatment counteracts those events (40). This is important because iNOS is one of the three NOS isoforms. It catalyzes the oxidative deamination of L-arginine to produce cytruline and nitric oxide (NO) and it is essential for immunity and vascular function. Moreover, it has been involved in the pathogenesis of various diseases through ROS/RNS induction. Indeed, ERK1/2 activation leads to increased iNOS and NO production (41, 42).

## Conclusions

The aforementioned studies report that GHRH induces ROS/RNS generation. GHRH antagonists can counteract those effects eliciting anti-inflammatory responses, which contribute to their anti-cancer activities. The exact mechanisms involved in those events are not completely understood, and are currently under investigation.

## Author contributions

AS: Conceptualization, Writing – original draft, Writing – review & editing. NB: Writing – review & editing.
